# 868. Early Organism Identification by Matrix Assisted Laser Desorption Ionization Time Of Flight Mass Spectrometry (MALDI TOF MS) Decreases the Time to Optimal Antibiotic Therapy for Common Bacterial Infections

**DOI:** 10.1093/ofid/ofad500.913

**Published:** 2023-11-27

**Authors:** Vishakh C Keri, Ankesh Gupta, Bimal Kumar Das, Sarita Mohapatra, Manish Soneja, Arti Kapil, Immaculata Xess, Naveet Wig

**Affiliations:** Wayne State University, Detroit, Michigan; All India Institute of Medical Sciences, New Delhi, New Delhi, Delhi, India; All India Institute of Medical Sciences, New Delhi, New Delhi, Delhi, India; All India Institute of Medical Sciences, New Delhi, New Delhi, Delhi, India; All India Institute Of Medical Sciences, Delhi, Delhi, India; All India Institute of Medical Sciences, New Delhi, New Delhi, Delhi, India; All India Institute of Medical Sciences, New Delhi, New Delhi, Delhi, India; All India Institute of Medical Sciences, DELHI, Delhi, India

## Abstract

**Background:**

MALDI-TOF helps in precise identification of microorganisms from positive cultures. It is based on the principle of proteomics and obviates the need for the use of biochemicals. It reduces the turnaround time for reporting microbiologic cultures. Early identification and reporting of the organism, before the availability of antimicrobial susceptibilities, may reduce the time to first antibiotic modification and impact the clinical outcomes of patients.

**Methods:**

This is a prospective observational two-arm comparative study conducted at a tertiary care centre in North India, with a sample size of 50 patients in the conventional arm and 100 patients in the interventional arm. The workflow of both arms are depicted in figure 1&2. The study population included patients in the medical ward and intensive care units (ICU) with positive cultures. The primary objective of this study was to compare the average time to first antibiotic modification post-culture positivity in the conventional and interventional arms and to compare clinical outcomes of patients in both arms. Escalation, de-escalation and any modification in antibiotics were also recorded.

**Figure:**
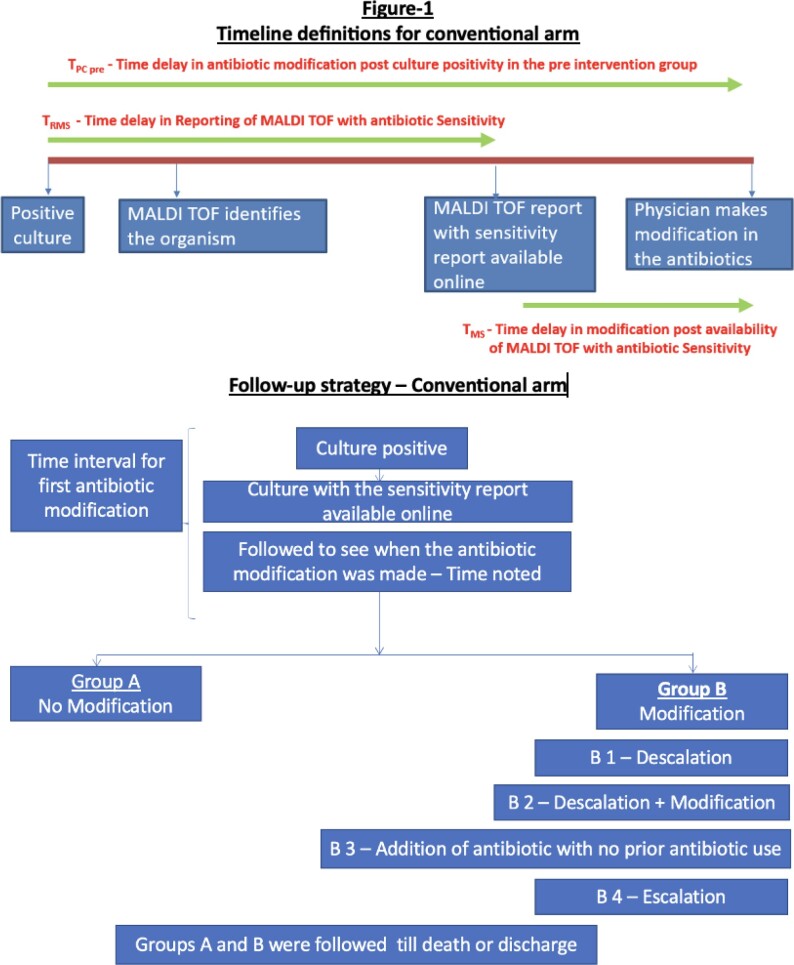

**Figure:**
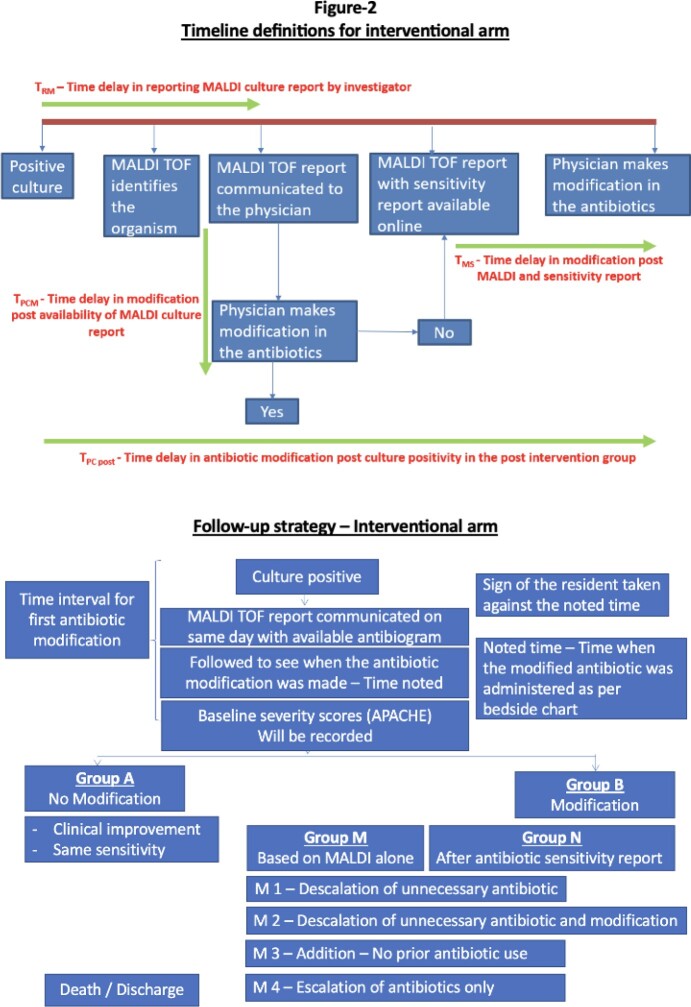

**Results:**

The median time to first antibiotic modification was significantly shorter in the intervention arm compared to the conventional arm (2010 mins vs 2905 mins, p=0.002). Overall 44/100 antibiotic modifications were made in the interventional arm out of which 43.2% (19/44) were made based on the MALDI report alone without awaiting susceptibilities. The major type of modification was the de-escalation of antibiotics (47.4%). Mortality rates were 32% in the conventional arm and 27% in the intervention arm, but the difference was not statistically significant (p=0.52) (table 1).

**Figure:**
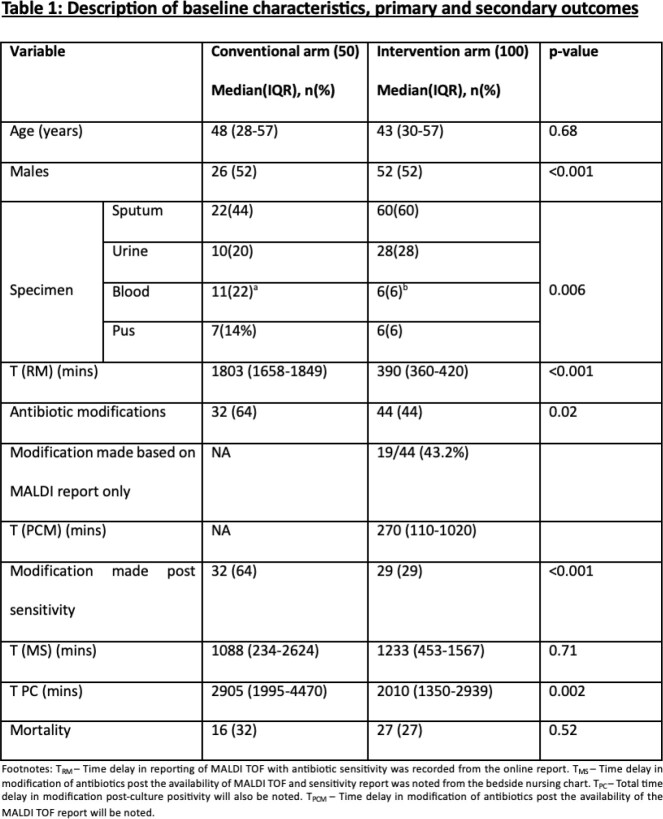

**Conclusion:**

MALDI-TOF helps in the early modification of antibiotics. The major utility is in the de-escalation of unnecessary antibiotics. Coupled with a robust antimicrobial stewardship programme, this modality can help in reducing antibiotic use.

**Disclosures:**

**All Authors**: No reported disclosures

